# Reaction and execution time rehabilitation among a child with CAS - single case study

**DOI:** 10.1192/j.eurpsy.2026.11695

**Published:** 2026-07-31

**Authors:** E. Vashdi, R. Popa

**Affiliations:** 1 Yaelcenter, Haifa, Israel; 2 Dinamic Equilibrium, Bucharest, Romania

## Abstract

**Introduction:**

Being non-verbal has a vast influence on child development and requires thorough rehabilitation process using significant tools and accuracy. It is similar to a non-mobile condition, but has more cognitive and emotional consequences. In this research we examine an advanced phase in helping a completely non-verbal child, 7 years old, to over come an obstacle on the way to spoken language. This child was able to acquire all syllables and hundreds of words under imitation in 3 years since he was 4 years old. However, the production time is very slow and requires many efforts. The research is examining techniques to reduce the reaction and execution time of words and syllables.

**Objectives:**

The objective was to test VML techniues for the improvement of reaction time in speech treatment for a child with developmental delay and apraxia of speech.

**Methods:**

Prospective Single case study design. We tested most single syllables in Dutch and a few word structures for typical execution time, reaction time, execution time, ratio between RT and expected RT, and ratio between CAS execution and typical one. Intervention based on VML method is designed for three months, afterwards a final test will take place.

**Results:**

RT was found elongated, about 5 times more then expected, and was growing with the length of structure usually. Execution time was found elongated, 4 times more for the CV and lower ratio for the words. The reason is probably elongated words production by the examiner which wasn’t typical.

**Conclusions:**

The elongated time for processing and execution damage the ability to use spoken language even when all sounds exist under imitation. The influence of this situation on daily life and rehabilitation into typical environment is significant and life changing.

At presentation we will present the full process and results, including sound samples.

**Disclosure of Interest:**

None Declared

